# Effects of Passive Leadership in the Digital Age

**DOI:** 10.3389/fpsyg.2021.701047

**Published:** 2021-08-26

**Authors:** Cheng-Hui Wang, Gloria H. W. Liu, Neil Chueh-An Lee

**Affiliations:** ^1^Executive Master of Business Administration Program, Da-Yeh University, Changhua, Taiwan; ^2^International Business School Suzhou, Xi'an Jiao Tong Liverpool University, Suzhou, China; ^3^Department of Marketing and Tourism Management, National Chiayi University, Chiayi, Taiwan

**Keywords:** social media, online emotional labor, passive leadership, job autonomy, resilience

## Abstract

Organizations must adapt to the trend of digitalization. Nowadays, social media engagement editors play an increasingly crucial role for organizational growth and prosperity in the digital age. Engagement editors are usually tasked to perform the functions of marketing, content production, and data analysis. They have to manage online communities on behalf of the organization, and encounter online audiences' frequent toxic and aggressive behaviors. Engagement editors thus are prone to emotional stress. Substantial literature has examined the influence of leadership style on employee performance. However, passive leadership is rarely studied. This research investigates (1) whether passive leadership would negatively affect engagement editors' performance (i.e., online interaction with audiences); and (2) how the negativity would be ameliorated by certain organizational policies (i.e., job autonomy) and their individual attributes (i.e., employee resilience) from the conservation of resource perspective. We surveyed 122 engagement editors and used the smartPLS 3.2.9 to analyze the data. This research provides important theoretical and practical implications.

## Introduction

The advance of digital technologies has ushered in digital transformation to many industries. One of the major changes is an emerging role, known as the engagement editor (Powers, 2015). Engagement editors are principally tasked to interact with online audience of an organization to promote the organization's digital presence, and ultimately, to increase its sales revenues or profits. The market demand for engagement editors is getting strong. In 2014, 80% of Fortune's Top 500 companies in the US have set up Facebook fan pages that are managed by social media engagement editors (Barnes and Lescault, 2014). This number climbs to 90% in 2017 (Clement, 2019).

Social media platforms afford organizations with two-way, real-time communication functionalities. Engagement editors thus can represent organizations to interact with their clients virtually, enabling organizations to get better insights about their clients' needs and expectations. However, cyberbullying is becoming a serious concern and becomes part of reality at work for engagement editors. In the face of netizens' verbal and emotional attacks, engagement editors are required to manage their feelings to express organizationally desired emotions (Grandey, 2000). This could be emotionally exhausted and requires substantial personal resources to cope with (Hochschild, 2012). Leadership can be an important source of resources that could support (or deplete) engagement editors.

The literature has identified different leadership styles (Bass and Avolio, 1990). Among them, passive leadership is the least studied (Eisenbeiß and Brodbeck, 2014). Passive leadership refers to a pattern of inaction and disengagement on the part of the management (Derue et al., 2011). Passive leadership is common in the workplace, with at least 20 percent of employees experiencing such practices by management (Aasland et al., 2010). However, issues related to passive leadership are often ignored. For example, Martinko et al. (2013) identify multiple types of abusive behaviors by supervisors, but do not recognize passive leadership as one of them. It is generally argued that the management does not intend to harm employees or organizations under most circumstances, and therefore passive leadership could not cause much harm to the organization (Barling and Frone, 2017).

In fact, under passive leadership, employees could suffer role overload, ambiguity, and conflict due to the lack of guidance and coordination (Hinkin and Schriesheim, 2008; Skogstad et al., 2014). Among the limited literature, Christie and Barling (2009) found that employees' long exposure to passive leadership could cause chronic stress, and difficulty regaining personal control of their work. Based on the perspective of conservation of resources (COR), we argue that leadership is an important factor that can act to facilitate or inhibit employees' resource gain to perform and to cope with their job demands and challenges (Hobfoll, 2002, 2011).

This study aims to study how passive leadership could negatively influence emotional labors and how the negativity of passive leadership can be ameliorated by both organizational and individual resources, i.e., job autonomy and employee resilience. We integrate literatures on leadership and work stress to develop our research model (Wang et al., 2019). We find that passive leaders can directly cause poor employee performance and indirectly cause it due to role overload perceived by employees. However, employee personal resource of resilience can help reduce the negativity of passive leadership. On the other hand, the organizational resource of job autonomy can negatively moderate the relationship between role overload and employee performance. This means that when job autonomy is high role overload does less harm to employee performance.

The remainder of the paper is organized as follows. The next section reviews the literature and develops a research model that outlines how passive leadership negatively influences engagement editors' performance and how job autonomy (i.e., organizational resource) and employee resilience (i.e., individual resource) help ameliorate the negativity. The method is described next and the results follow. The paper ends with a discussion of the results and implications for research and practice.

## Background Literature

Social media have become the most commonly used communication channels for contemporary organizations. Increasing organizations employ social media to build or maintain both online and offline relationship with their clients. The news industry is no exception. It is found that news organizations use social media to build and maintain a huge fan/follower base (PewResearchCenter, 2010). Indeed, people, especially the millennial generation, increasingly rely on their social networks to access information and news (Hermida et al., 2012; Mitchell et al., 2013).

Technological functionalities of social media enable two-way interaction between audiences and news organizations. For example, audiences can comment, like, share, or even co-create news content on social media platforms (e.g., via uploading videos or photos). Another example is the function of tagging. Tagging allows the archival of specific knowledge domains, and facilitates the collective reuse of said knowledge (Majchrzak et al., 2013). Audience participation enabled by social media has disrupted the long-established professional norms of journalism which emphasizes the gatekeeping and agenda-setting role of journalism (Heinonen, 2011). Many seasoned journalists express difficulty in adapting to new practices associated with social media, including managing online communities for effective news production and dissemination (Kenney et al., 2000).

News organizations increasingly see their audiences as active participants. The role of engagement editors is thus created to exploit social media and manage their active audiences to news organizations' benefits. Unlike traditional journalists, engagement editors mainly work virtually. Their daily job may include monitoring and analyzing website traffic, interacting with their digital audiences, and organizing online/offline activities to further engage their audiences (Kenski and Stroud, 2006; Lin, 2006).

The most important task of engagement editors is to interact and maintain good relationship with fans and followers virtually. Engagement editors are thus required to have professional knowledge of journalism, and demonstrate empathy and excellent negotiation skills to manage customer relationships (Aldoory, 2005; L'Etang and Pieczka, 2006). However, to meet their job requirements, they often have to bear the brunt of their audience's reaction, stage inauthentic feelings, or even learn to please fans (Phillips and Young, 2009). This can easily deplete engagement editors' personal resources.

## Conservation of Resources Theory

According to the conservation of resources theory (COR), performing tasks and coping with stressful situations require personal resources, such as time, physical energy, emotional energy, or attention (Hobfoll, 1988, 2001, 2002). However, these resources are limited. Individuals thus will strive to acquire and maintain their personal resources to continuously meet their job requirements, such as via rest or food intake. When sufficiently drained, individuals will not be able to perform. Therefore, if the organization can provide resources or support, it will help replenish or protect employees' personal resources and thus increase their work outputs. For example, organizations can create a friendly and collaborative work environment that is conducive to the gain or protection of employees' personal resources or the avoidance of the loss of valued resources.

### Passive Leadership

Passive leaders ignore their responsibilities and do not empower employees (Hamidifar, 2014). They do not deal with employee issues and workplace problems until it is too late. This makes it almost impossible to foster or reinforce appropriate behavior at the workplace (Derue et al., 2011). Passive leaders thus are likely to cause employee confusion, role conflict, workplace bullying and uncivil behaviors, resulting in psychological distress, work fatigue and burnout (Skogstad et al., 2007; Harold and Holtz, 2015). Further, passive leaders can reduce employees' trust and create unfair feelings (Holtz and Hu, 2017). They can bring significant harm, especially to employees' roles and conflicts, well-being, work attitude, and organizational commitment (Judge and Piccolo, 2004; Derue et al., 2011; Zineldin and Hytter, 2012; Jackson et al., 2013; Skogstad et al., 2014; Buch et al., 2015).

Given that the engagement editor is an emerging category of role in organizations, job descriptions, rules and role expectations are not fully developed yet. This can create stressful situations. Passive leaders could further deplete or drain engagement editors' personal resources by causing more confusion, conflicts and mistrust. As engagement editors lose control of their personal resources, they will not be able to perform their jobs and reduce interaction with fans to conserve personal resources. Therefore, we propose that:

Hypothesis 1: Passive leadership will reduce engagement editors' online interaction with fans and followers.

### Role Overload

Employees experience role overload when job demands and responsibilities exceed their abilities (Bolino and Turnley, 2005). Role overload is closely related to work stress as employees are pressured by too much responsibility and commitments (Brown et al., 2005). Role overload thus could trigger negative attitudes in employees, reduce job performance and organizational commitment, and cause absence (Rodell and Judge, 2009; Jensen et al., 2013). Role overload is more salient a stressor than role ambiguity and conflict (Narayanan et al., 1999; Mulki et al., 2008; Gurbuz et al., 2013).

Employees must fulfill the demands of both their job and organizational roles to meet the expectations of the organization (Welbourne et al., 1998). Employees on the frontline must also satisfy the needs of customers (Crawford et al., 2010; Chiu et al., 2015). Role overload thus could come from diverse sources (Mulki et al., 2006).

With the advance of digital technologies, the highly interactive, real-time online environment forces front-line employees, such as engagement editors, to shoulder more responsibilities and suffers more role overload as they have to meet expectations of diverse stakeholders within a short timeframe (e.g., customers) (Itani and Inyang, 2015; Yang et al., 2015). For example, when pieces of sensitive news are broadcasted, some audiences or online haters may react irrationally and bombard engagement editors with unfriendly messages or even personal attacks. However, passive leaders tend to turn a blind eye to the situation and leave engagement editors to deal with those haters by themselves. Without a proper role script to follow, this thus increases engagement editors' workload because they have to explore methods to handle those issues and at the same time, to avoid punishment. Therefore, we propose that:

Hypothesis 2: Passive leadership will increase engagement editors' role workload.

For engagement editors, role overload would negatively affect their online interaction with fans. The online interaction defines customer experiences with the organization engagement editors represent. This may involve engagement editors' attitudes and behaviors toward fans and the interactive formats adopted (Karatepe et al., 2005). Quality online interaction requires mutual trust between engagement editors and customers (Ekinci and Dawes, 2009). Role overload depletes engagement editors' cognitive and emotional resources, such as empathy and kindness. They thus will not be able to behave compassionately, helpfully and professionally (Jha et al., 2017). Therefore, we propose that:

Hypothesis 3: Role overload will reduce engagement editors' online interaction with fans and followers.

Passive leaders keep supervisor-employee interaction to a minimum, as they would not provide employees with instruction, feedback, or support (Skogstad et al., 2007; Buch et al., 2014). They are both unethical and uncaring, and can easily trigger negative emotions and bring about stress among engagement editors (Einarsen et al., 2007). Since passive leaders rarely involve in task planning or interacting with their subordinates, they can bring about more stress and aggravate engagement editors' already demanding work, leading to more role overload in engagement editors (Skogstad et al., 2007; Buch et al., 2014; Barling and Frone, 2017; Vullinghs et al., 2018). Consequently, engagement editors would reduce interaction with fans to preserve their personal resources. Therefore, we propose that:

Hypothesis 4: Role overload will mediate the negative relationship between passive leadership and engagement editors' online interaction with fans and followers.

### Job Autonomy

Job autonomy refers to employees' discretionary power that is granted by organizations to perform tasks at their own pace and in their own ways. Therefore, an employee can choose to not follow the prescribed working schedule, and has the power to make discretionary decisions about how they will execute their jobs (Ilies et al., 2005). Job autonomy is particularly important in a highly variable work environment (Troyer et al., 2000). This is because job autonomy grants employees the freedom to decide how they would like to complete tasks and to adapt to the changing work environment. Job autonomy thus has a positive impact on performance, creativity, and knowledge sharing (Morgeson et al., 2005; Pee and Lee, 2015; Llopis and Foss, 2016), and can reduce job uncertainty (Idaszak and Drasgow, 1987).

Job autonomy is a crucial organizational resource that helps alleviate the negative effects of work stress (Abraham, 2000; Grandey et al., 2005; Goussinsky, 2011). Job autonomy allows employees to effectively deploy and seek resources themselves. When there is low job autonomy, mistakes or errors are less tolerated (Fuller et al., 2010; Liu et al., 2011). The negative effect of role overload thus is likely to be reinforced. Conversely, when employees have job autonomy, they are more likely to learn from their mistakes and errors without being punished (Dierdorff and Morgeson, 2007; Liu et al., 2011). As engagement editors work with high role overload, job autonomy not only reduces perceived work stress, but also creates a fault-tolerant space, thereby motivating engagement editors to adapt their behaviors to interact with fans. We thus propose:

Hypothesis 5: Job autonomy will negatively moderate the relationship between role overload and engagement editors' online interaction with fans and followers.

### Resilience

Workplace pressure is inevitable. Yet, individuals react to pressure differently. Resilient people, after a brief interruption by a tense situation, can return to normal and maintain their mental health (Luthar et al., 2000; Bonanno, 2005). American Psychological Association defines resilience as “adaptation to adversity, trauma, sources of significant stress, such as family and relationship issues, health issues or workplace and financial stress.” (Southwick et al., 2017) defined resilience as the ability to regain balance following exposure to adverse events. In the face of high-pressure situations, resilient people can develop a response strategy and resist the adverse conditions to construct the future. Masten et al. (1990) emphasize that resilience is a dynamic process in which individuals interact with the environment (Norris et al., 2008) also argue that resilience is a potential outcome after employee survive stressful events. Therefore, resilience is not only a psychological trait that leads to positive outcomes, but also can be altered and cultivated (Robertson et al., 2015).

Management can play an important role to help enhance employee resilience, such as via setting a role model (Grotberg, 2003). However, passive leaders tend to set a role model who avoids and ignores problems, and evade their responsibilities. Over time, employees thus develop a similar coping strategy, making them prone to more stress and helplessness in the face of problems. Therefore, we hypothesize:

Hypothesis 6: Passive leadership will reduce engagement editors' resilience.

Resilient people are more likely to adapt to changes to achieve high job performance, despite adverse conditions such as long working hours, poor working conditions, and complex and challenging environments (Sonnenfeld and Ward, 2008; Carucci, 2017). Resilient people are better at regulating their own emotion, controlling impulse and adjusting goals; they are also more likely to demonstrate empathy, pragmatic optimism and high self-efficacy (Mourlane, 2020). With high resilience, engagement editors thus can maintain quality interaction with fans, regardless of the sometimes toxic online environment. Therefore, we hypothesize:

Hypothesis 7: Engagement editors' resilience will increase their online interaction with fans and followers.

Resilience can be seen as one kind of personal resources that individuals can use consciously to create positive emotions (Handley et al., 2004) and change their thoughts and behaviors (Masten, 2001; Tugade and Fredrickson, 2004). Therefore, adverse conditions, such as time pressure or demanding work responsibility, can be reinterpreted as either a challenge or a threat. Resilient individuals will take those conditions as a challenge and increase their inputs or commitment to overcome the challenge (Crawford et al., 2010). Over time, they can turn such conscious strategizing into an automated process (Bargh and Chartrand, 1999). Whenever difficult situations occur, resilient people can easily initiate positive responses (Isen and Diamond, 1989). In contrast, adverse conditions are likely to induce negative emotions, such as fear, anxiety and anger within individuals lacking resilience, further depleting their personal resources and leading to their reduction in work commitments (Erez and Isen, 2002; May et al., 2004).

When supervised by a passive leader, engagement editors face the dilemma of job demand being greater than resources. Resilient engagement editors are likely to initiate positive emotions and look for other sources for replenishing personal resources (e.g., peers, friends). They thus can prevent depletion of personal resources and maintain quality interaction with fans. In contrast, without resilience, engagement editors will have to simultaneously deal with the passive leader, its attendant negative emotion and stress, and job demands. This thus could drain their personal resources to the point that they feel burnout. We thus hypothesize:

Hypothesis 8: Resilience will mediate the relationship between passive leadership and engagement editors' online interaction with fans and followers.

**Figure 1:**
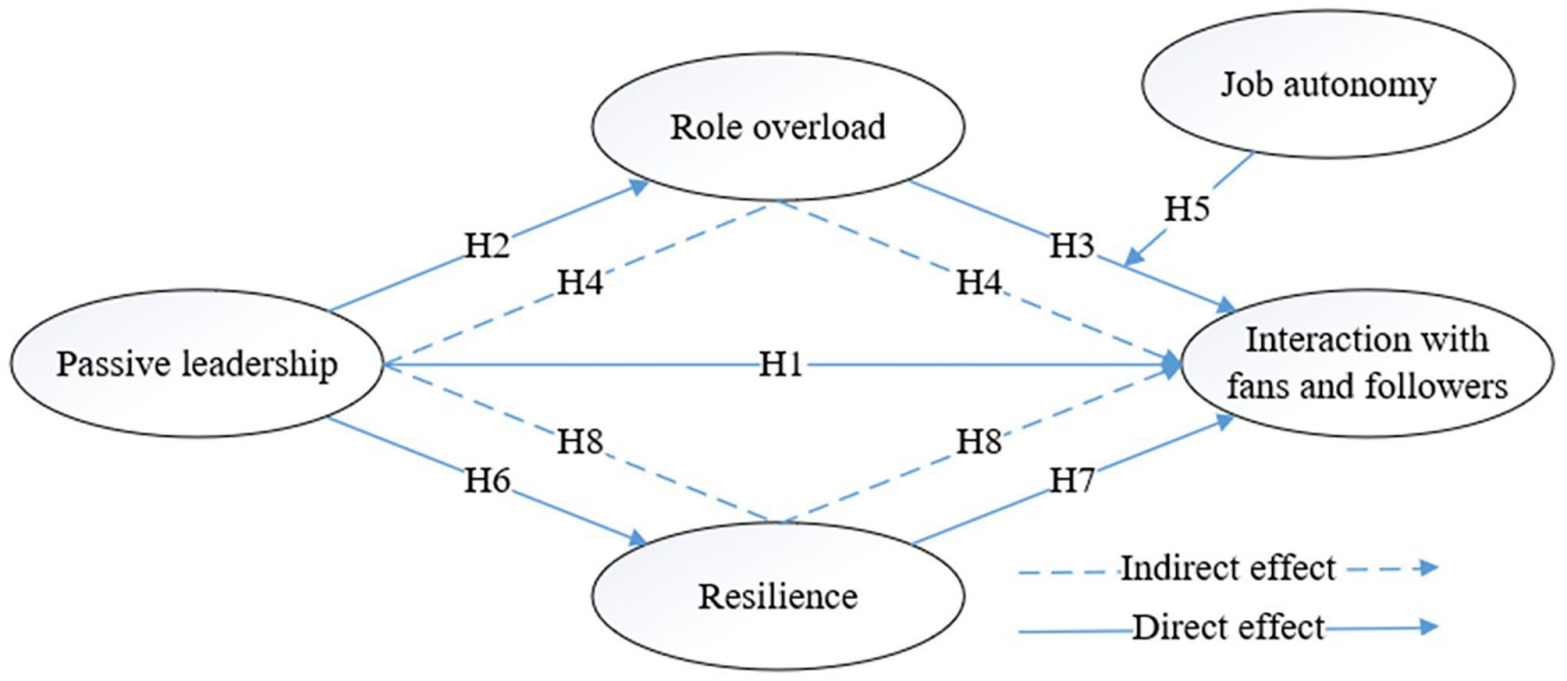
Research model.

Our research model is exhibited in Figure 1.

## Methodology

A survey questionnaire was administered to collect data. Our measures were principally derived from existing scales and adapted to suit the research context. To better comprehend social media engagement editors' work context and process, we also interviewed 2 engagement editors and 3 managers in a social media news organization in Taiwan. After compiling an English version of the questionnaire, we translated the survey items into Chinese. Two bilingual scholars next verified and refined the translation accuracy of the survey items. The Chinese version of the draft questionnaire was then pretested with 2 senior news editors for examining its face and content validity, resulting in modification of the wording of some items. We operationalized all constructs using multi-item reflective measures with a five-point Likert scale anchored from “strongly disagree” to “strongly agree.” The measures are discussed below and shown in Appendix A.

We measured *passive leadership* using four items to assess the extent to which individual engagement editors perceive that supervisors withdraw from making decisions and managerial activities (Kelloway et al., 2006). *Job autonomy* was measured by four items which assess the extent to which individual engagement editors have discretion to make decisions about their work (Breaugh, 1985). *Role overload* was measure by three items that are adapted from Bolino and Turnley (2005), assessing the extent to which employees feel that there are too many responsibilities or activities expected of them in light of the time available, their abilities, and other constraints. *Resilience* was measured by four items adapted from Stephens et al. (2013). We assessed *online interaction with fans and followers* with four items adapted from Liu (2003). Finally, we control *years in managing online communities* that may influence the levels of interactivity.

### Sample and Data Collection

A cross-sectional survey for social media engagement editors was administrated to collect data from the top 30 online news firms based on the Year 2018 of the Top sites in Taiwan, published by Alexa Internet, Inc. We first emailed the top managers of these firms to obtain permission to survey their social media engagement editors. Nineteen firms, including top 1 to top 11, allowed us to distribute questionnaire to their engagement editors. We finally distributed our questionnaire to all their 200 social media engagement editors and collected their responses in person. One hundred and twenty-two responses were returned and valid for subsequent analysis. This yielded an effective response rate of 61%. Table 1 exhibits the characteristics of the sample. Among the respondents, 71% were <30 years old, and 70% had experiences in managing online communities for <3 years. Our data demonstrate that social media engagement editing is still in its adolescence in the news industry in Taiwan, given most respondents are relatively junior and young.

**Table 1:** Profile of the respondents (*N* = 122).

	**No**.	**%**
**Gender**		
Male	43	35
Female	79	65
**Ages**		
20–25	43	35
26–30	44	36
31-35	22	18
36–40	6	5
40 above	7	6
**Year(s) of experience in managing online communities**		
<1 year	24	20
1–3 years	61	50
3–5 years	26	21
5–7 years	8	7
More than 7 years	3	2

### Data Analysis

We conducted a PLS structural equation model (PLS–SEM) testing to validate our measures and test hypotheses. The software used is SmartPLS Version 3.2.9.

#### Assessment of Common Method Variance

To assess common method variance (CMV), we conducted a Harmon's single-factor test (Podsakoff et al., 2003). As expected, we were able to extract four factors with eigenvalues of >1 which collectively accounted for 68.82% of the variance in the data, with the first factor accounting for 37.07% of said variance. This demonstrates that CMV is not a serious concern. We also incorporated a latent marker variable (MLMV) in our survey to correct for CMV when using PLS (Chin et al., 2012). This approach requires collecting multiple items that have no nomological relationship with the research items. We followed the guidelines introduced by Chin et al. (2012) and selected the items used to measure “trying new features” in Microsoft Office (Sun, 2012) as the MLMV. We then could conduct the construct level correction (CLC) to partial out the CMV effects at the structural model (Chin et al., 2012). CLC involves creating as many CMV control constructs as there are constructs in the research model. Each CMV control uses the same entire set of MLMV items. The CMV construct was modeled as impacting each model construct. As such, more accurate estimates of the structural paths can be obtained (Chin et al., 2012).

#### Measurement Model Evaluation

We assessed construct validity and reliability according to the guidelines by Henseler et al. (2016) and Hair et al. (2017). Outer loadings for all items were higher than 0.7 and were significant at 1% level except for four items (please see Appendix A). We delete the items of passive leadership and job autonomy, but we only delete the worse loading item (0.57) and keep the better loading item (0.617) of resilience in order to keep content validity (Hair et al., 2017). The rho_A, composite reliability (CR) and Cronbach's alpha estimates, reported in Table 2, were above 0.7 for all constructs, indicating good internal consistency and the reliability of the scales (Henseler et al., 2016; Hair et al., 2017), except for resilience (Cronbach's alpha = 0.6; rho_A = 0.63). Considering the exploration nature of this study, such values of Cronbach's alpha and rho_A are acceptable. We further assessed the convergent validity of our constructs based on average variance extracted (AVE). The AVE of each construct exceeded the minimum threshold value of 0.5 (Henseler et al., 2016; Hair et al., 2017). The combined results demonstrated sufficient convergent validity of the constructs.

**Table 2:** Inter-construct correlations, reliability measures, and HTMT (*N* = 122).

**Construct**	**Items**	**Cronbach's α**	**ρ_A**	**CR**.	**AVE**.	**1**	**2**	**3**	**4**	**5**
Passive leadership	3	0.9	0.9	0.94	0.84	**0.92**				
Job autonomy	3	0.72	0.75	0.84	0.63	−0.14 (0.25; [0.1, 0.49])	**0.79**			
Role overload	3	0.85	0.87	0.91	0.77	0.44 (0.50; [0.30,0.68])	−0.17 (0.25; [0.09,0.57])	**0.88**		
Resilience	3	0.6	0.63	0.79	0.56	−0.23 (0.32; [0.14, 0.58])	0.43 (0.66; [0.34, 0.69])	0.05 (0.17; [0.12, 0.48])	**0.75**	
Interaction with fans and followers	4	0.83	0.85	0.89	0.66	−0.36 (0.42; [0.22, 0.60])	0.1 (0.14; [0.09, 0.34])	−0.19 (0.21; [0.12, 0.43])	0.31 (0.42; [0.20, 0.70])	**0.82**

*Note: (1) Square roots of average variance extracted are shown on the diagonal with bold font; (2) HTMT and their 95% CI are shown in parentheses (HTMT; 95% CI, two-tailed test); (3) The 95% CI of HTMT are estimated by 5,000 bias-corrected and accelerated bootstrapping with confidence intervals bias corrected (Henseler et al., 2016)*.

Discriminant validity is established when (1) items load more highly on the construct that they are intended to measure than on other constructs, (2) the square root of the AVE by each construct is larger than the inter-construct correlations (Hair et al., 2017), and (3) heterotrait-monotrait ratio of correlation (HTMT) is significantly smaller than 1. Cross-loadings were computed by calculating the correlations between a latent variable's component scores and the manifest indicators of other latent constructs (Hair et al., 2017). Without exception, all items loaded more highly on their own construct than on other constructs (see Appendix B). As shown in Table 2, the square root of the AVE for each construct was greater than the correlations between the construct and other constructs, indicating that all the constructs shared more variances with their indicators than with other constructs. All HTMT values, also shown in Table 2, were significantly smaller than 1 with 95% CI, indicating clear distinction between two constructs. Thus, our measures exhibited sufficient discriminant validity.

#### Structural Model

We first assessed multi-collinearity by examining each set of predictor constructs separately for each subpart of the research model (Hair et al., 2017). In our model, all the variance inflation factors (VIF) of endogenous constructs are <1.71 which is well below the cutoff value of 5 (Hair et al., 2017), indicating no multi-collinearity problem in our model.

**Figure 2:**
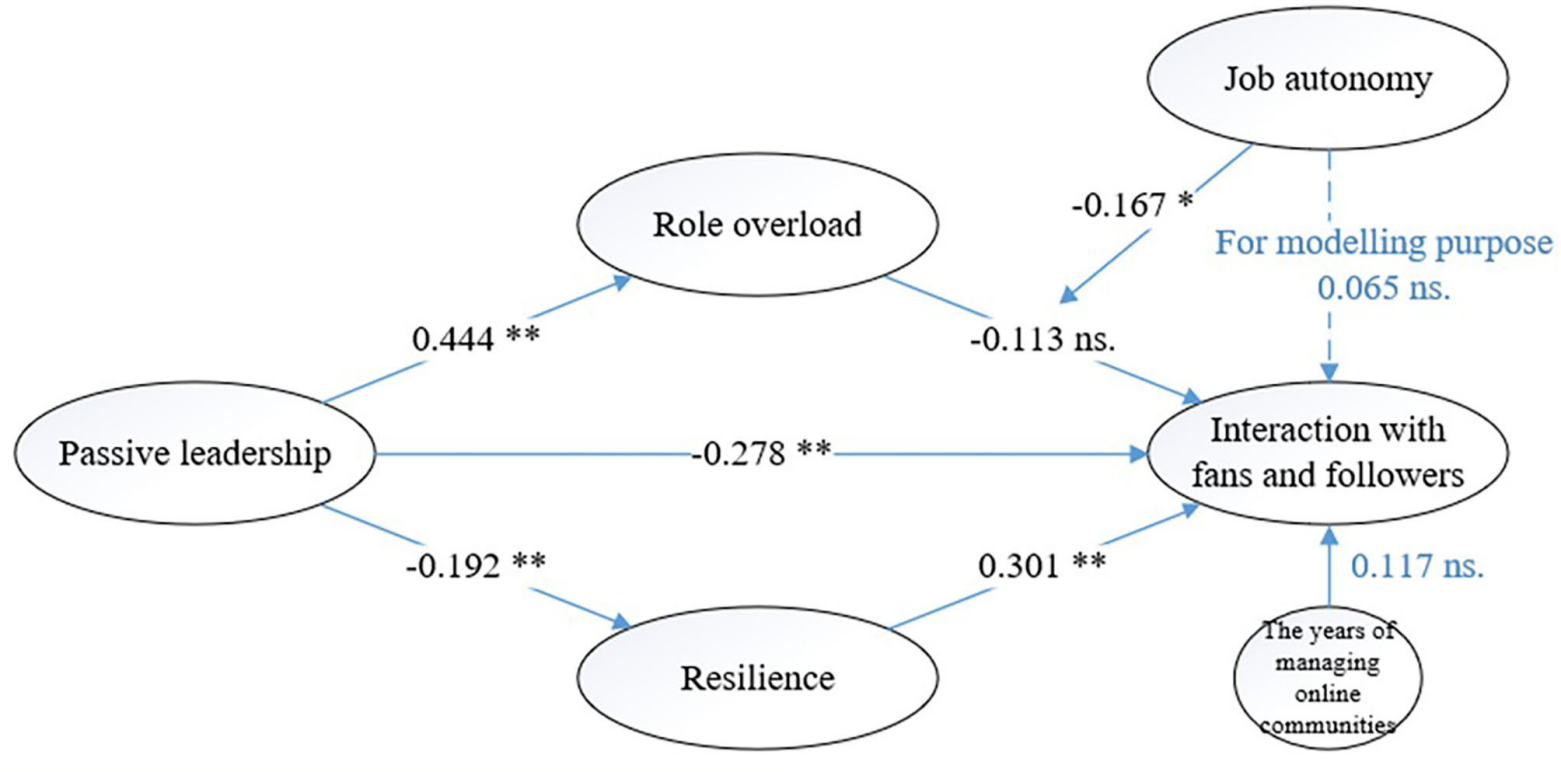
Results of the structural model. We hide the MLMV construct in order to simplify the figures. NS, Non-significant; *p < 0.05; **p < 0.01 (one-tailed test).

To assess the significance of the path coefficients in the inner model, SmartPLS was applied to generate 10,000 samples using a bootstrapping technique with the PLS algorithm, no sign changes, a path weighting scheme, and a bias-corrected and accelerated CI. We also use Lohmoeller settings for initial weights (Hair et al., 2017). We applied the two-stage approach to create the interaction term with the standardized approach suggested by Hair et al. (2017) for testing the moderating effects. The full model has an *R*^2^ of 27.4% for the interaction with fans and followers, 19.6 for role overload, and 27.5% for resilience. Figure 2 shows the results of structural model estimation.

We first examined the direct effects of our research model. The results show that passive leadership has significant negative effect on the interaction with fans and followers (support H1: *p* < 0.01). Our analysis also reveals that passive leadership positively influences role overload (support H2: *p* < 0.01), but negatively influences resilience (support H6: *p* < 0.01). While role overload does not reduce the interaction with fans and followers (reject H3: p>0.05), resilience positively affects the interaction with fans and followers (support H7: *p* < 0.01). Job autonomy negatively moderates the relationship between role overload and the interaction with fans and followers (support H5: *p* < 0.05). We further compared the *R*^2^ of the models with and without the moderator (i.e., 27.4% & 22%) to calculate *f* ^2^ to determine the effect size of the moderator (i.e., job autonomy). We find that *f* ^2^ is 0.0744, indicating a small to medium effect (the values of 0.02, 0.15, and 0.35, respectively, represent small, medium, and large effects) (Hair et al., 2017). Finally, the effect of the control variable on interaction is insignificant (i.e., the years of managing online communities).

To test mediating effect of role overload and resilience, we followed the guidelines suggested by Zhao et al. (2010) for justifying full or partial mediation: we conducted the mediation regression method with bias corrected bootstrap approach for examining the significance of indirect paths. We then adopted the simple mediation model (Preacher and Hayes, 2004; Hayes, 2013) to test the indirect paths with single mediators in the model. Because these approaches are regression based, we used PLS algorithm to obtain latent variables of the research constructs as inputs for performing the mediation regression method and 5,000 resampling on SPSS macros provided by Hayes (2013). Based on these procedures, all indirect paths can be tested reliably and validly.

Table 3 summarizes the results of the mediation model. As suggested by Zhao et al. (2010), we first examined the significance of indirect effects. The results indicate that the indirect effect of passive leadership on the interaction with fans and followers through role overload is insignificant at *p* > 0.05 level since zero is included in the 95% confidence interval (CI). The indirect effect of passive leadership on the interaction with fans and followers through resilience is significant at *p* < 0.05 level since zero is excluded in the 95% CI. We then examined the significance of direct effect from independent variable to dependent variable with the mediator controlled in order to justify full or partial mediation (see column c' in Table 3). Consequently, while resilience partially mediates the relationship between passive leadership and the interaction with fans and followers (support H8), role overload fails to ameliorate the negative relationship between passive leadership and the interaction with fans and followers (reject H4).

**Table 3:** Significance of single-mediator paths.

**Row**	**Path**	**c**	**α**	**β**	**c'**	**αβ**	**Bootstrap 95% CI**	**Type**
1	Passive leadership -> role overload -> interaction	−0.35 (0.00)	0.44 (0.00)	−0.04 (0.70)	−0.35 (0.00)	0.02	−0.11, 0.11	No mediation
2	Passive leadership -> resilience -> interaction	−0.31 (0.00)	−0.24 (0.00)	0.24 (0.00)	−0.31 (0.00)	−0.05	−0.14, −0.01	Partial mediation

*c, the total effect of independent variable on dependent variable; α, the effect of independent variable on mediating variable; β, the effect of mediating variable on dependent variable when controlled independent variable; c', the effect of independent variable on dependent variable when controlled mediating variable t values shown in parenthesis*.

## Implications and Conclusion

Interaction with fans is among the major tasks of engagement editors. The results show that passive leadership reduces engagement editors' interaction with fans. Prior research focused on negative impacts of passive leadership on internal organization, such as unclear roles, employee well-being, work attitude and organizational commitment (Zineldin and Hytter, 2012; Skogstad et al., 2014; Buch et al., 2015). Our study provides support for more negative effects of passive leadership, including role overload and reduced interaction with fans/customers. The reduced interaction with fans/customers indicates that passive leadership can create a spillover effect beyond organizational boundaries in the digital age, i.e., by reducing employees' online interaction with fans and followers. In the case of the engagement editor on social media platforms, interaction with fans create a public discourse that, to some extent, projects the organization's image and position to their stakeholders. If supervisors withdraw from their managerial duties, employees would be both physically and mentally exhausted, leading to reduced online interaction with clients and poor projection of the organizational image.

This study also demonstrates that resilience is effective in alleviating negative impacts of passive leadership. Resilient employees tend to see negative factors as challenges. They thus strive to overcome impediments when interacting with fans. Indeed, emotional workers, such as engagement editors and frontline employees, have to bear the brunt of their clients/fans, their job thus can be highly emotion-taxing and requires the development of a customer-oriented attitude and a strong concern for their customers (Yoo and Arnold, 2016). Further, because resilience predisposes employees to interpret negative factors in a positive light, resilience is more likely to foster positive emotions and thoughts in employees, leading to more resources gained. The initial resource (i.e., resilience) thus may beget further resource gain, leading to “gain spirals” (Hobfoll, 2001). Future studies may examine whether and how resilience facilitates employees to gain more resources. In practice, organizations can enhance employee resilience through human management measures, including training on emotional management, enhanced self-esteem, internal reflection and problem-solving skills (Ungar, 2004). As a result, employees can automate the positive reaction and emotional regulation when encountering difficult customers or stakeholders online.

In addition, job autonomy, one kind of resources, can reduce the stress induced by excessive organizational control and monitoring (Holman et al., 2002). Job autonomy is particularly important for the online environment due to it being highly unpredictable and dynamic. When facing abusive customers, job autonomy allows engagement editors to choose appropriate responses and reduce emotional dissonance between their real emotions and organizationally desired ones (Abraham, 2000). Therefore, job autonomy is particularly important because all situational contingencies cannot be designed in organizational control. For example, when there has been an attack occurring, any missteps taken by engagement editors may incur even more attacks. The easiest way is thus to stop responding to save personal resources (i.e., organizationally undesired response). To prevent engagement editors' undesired passive reaction like this or “surface acting” (e.g., a cynical or detached response to fans or followers) (Yoo and Arnold, 2016), organizations can allow job autonomy with enabling control to ensure that employees self-goals are consistent with the organizational goals (i.e., saving personal resources as well as effective online interaction) (Adler and Borys, 1996; Liu and Chua, 2020). Enabling control involves the provision to employees of contingent information and the right to make decisions themselves. As such, employees can feel safe to take creative, effective action independently or in collaboration with each other (Liu and Chua, 2020). Employees thus would not act at the cost of organizational benefits (e.g., reduce performance to save personal resources).

Finally, the strong direct effect of passive leadership on online interaction after considering the mediation effects of role overload and employee resilience suggests that there might be other unidentified factors that mediate the relationship between passive leadership and online employee performance (Preacher and Hayes, 2008). For example, mistrust and unfair feelings (Holtz and Hu, 2017) may be potential mediators that influence online interaction. More research is needed to uncover various routes through which passive leadership leads to decreased performance.

This study has some limitations. First, this study conducted a cross-sectional survey. Our conclusion thus is only tentative. Second, this paper adopts perceptual measures that may not accurately reflect the true relationships between the constructs. We thus conducted a Harmon's single-factor test which indicates this limitation is not serious, and we also included a marker variable to partial out CMV. Third, we only study social media engagement editors in the news industry. Future studies may consider other industries with a similar focus on online customer engagement (e.g., online retailing). This may enhance generalizability of our results.

## Data Availability Statement

The original contributions presented in the study are included in the article/supplementary material, further inquiries can be directed to the corresponding author/s.

## Ethics Statement

The ethics sanction of this study was granted by the Human Research Ethics Committee at National Cheng Kung University in Taiwan (NO: 109-506). The participants provided their written informed consent to participate in this study.

## Author Contributions

All authors listed have made a substantial, direct and intellectual contribution to the work, and approved it for publication.

## Conflict of Interest

The authors declare that the research was conducted in the absence of any commercial or financial relationships that could be construed as a potential conflict of interest.

## Publisher's Note

All claims expressed in this article are solely those of the authors and do not necessarily represent those of their affiliated organizations, or those of the publisher, the editors and the reviewers. Any product that may be evaluated in this article, or claim that may be made by its manufacturer, is not guaranteed or endorsed by the publisher.

## Appendix A. Measurement Items

**Table:**

**Constructs**	**Measurement items**	**References**
Passive leadership	• My supervisor… 1 Spends his/her time trying to 'put out fires'. 2 Fails to intervene until problems become serious. 3 Fails to follow-up requests for assistance. •*Makes clear what I can expect to receive, if my performance meets designated standards. (deleted)*.	Kelloway et al., 2006
Job autonomy	1 I am free to decide how to go about getting my work done. 2 I am able to decide for myself what my objectives are. 3 I am able to affect other people's evaluation on me, so that I know how to emphasize the importance of myself. •*I am able to decide for myself when to do my work. (deleted.)*	Breaugh, 1985
Role overload	1 The amount of work I am expected to do is too great. 2 I never seem to have enough time to get everything done at work. 3 It often seems like I have too much work for one person to do.	Bolino and Turnley, 2005
Resilience	1 I am getting better at my work because I learn from my mistakes. 2 I see challenges as an opportunity to learn. 3 I find ways to handle unexpected situations. •*Dealing with difficult colleagues (or situations) enables me to grow. (deleted)*.	Stephens et al., 2013
Online interaction with fans and followers	1 Our fan page is effective in gathering visitors' feedback. 2 Our fan page facilitates two-way communication between itself and the visitors. 3 Our fan page listens to visitors. 4 Our fan page gives visitors the opportunity to talk back.	Liu, 2003

## Appendix B. Cross Loadings

**Table:**

	**Passive leadership (PL)**	**Job autonomy (JA)**	**Role overload (RO)**	**Resilience (RE)**	**Online interaction with fans and followers (OI)**
PL1	**0.92**	−0.08	0.38	−0.21	−0.35
PL2	**0.93**	−0.08	0.38	−0.17	−0.34
PL3	**0.89**	−0.23	0.45	−0.23	−0.31
JA1	−0.17	**0.74**	−0.23	0.18	0.05
JA2	−0.25	**0.82**	−0.22	0.41	0.08
JA3	0.02	**0.82**	−0.02	0.37	0.09
RO1	0.42	−0.17	**0.88**	−0.01	−0.17
RO2	0.35	−0.14	**0.84**	0.07	−0.05
RO3	0.39	−0.14	**0.91**	0.08	−0.24
RE1	−0.22	0.41	−0.09	**0.6**	0.13
RE2	−0.19	0.27	0.04	**0.78**	0.3
RE3	−0.12	0.31	0.13	**0.84**	0.24
OI1	−0.31	0.03	−0.2	0.23	**0.83**
OI2	−0.25	0.05	−0.08	0.21	**0.84**
OI3	−0.33	0.09	−0.14	0.32	**0.85**
OI4	−0.27	0.18	−0.19	0.22	**0.73**
